# Supplementary material for: Interleukin 10 controls the balance between tolerance, pathogen elimination, and immunopathology in birds
Source: eLife. 2025 Oct 16;14:RP106252. doi: 10.7554/eLife.106252 (PMC12530801; doi:10.7554/eLife.106252)
Supplement: Figure 1—figure supplement 4—source data 1. [file elife-106252-fig1-figsupp4-data1.zip › Figure 1-figure supplement 4-source data 1/Figure 1-figure supplement 4-source data 1.pdf]

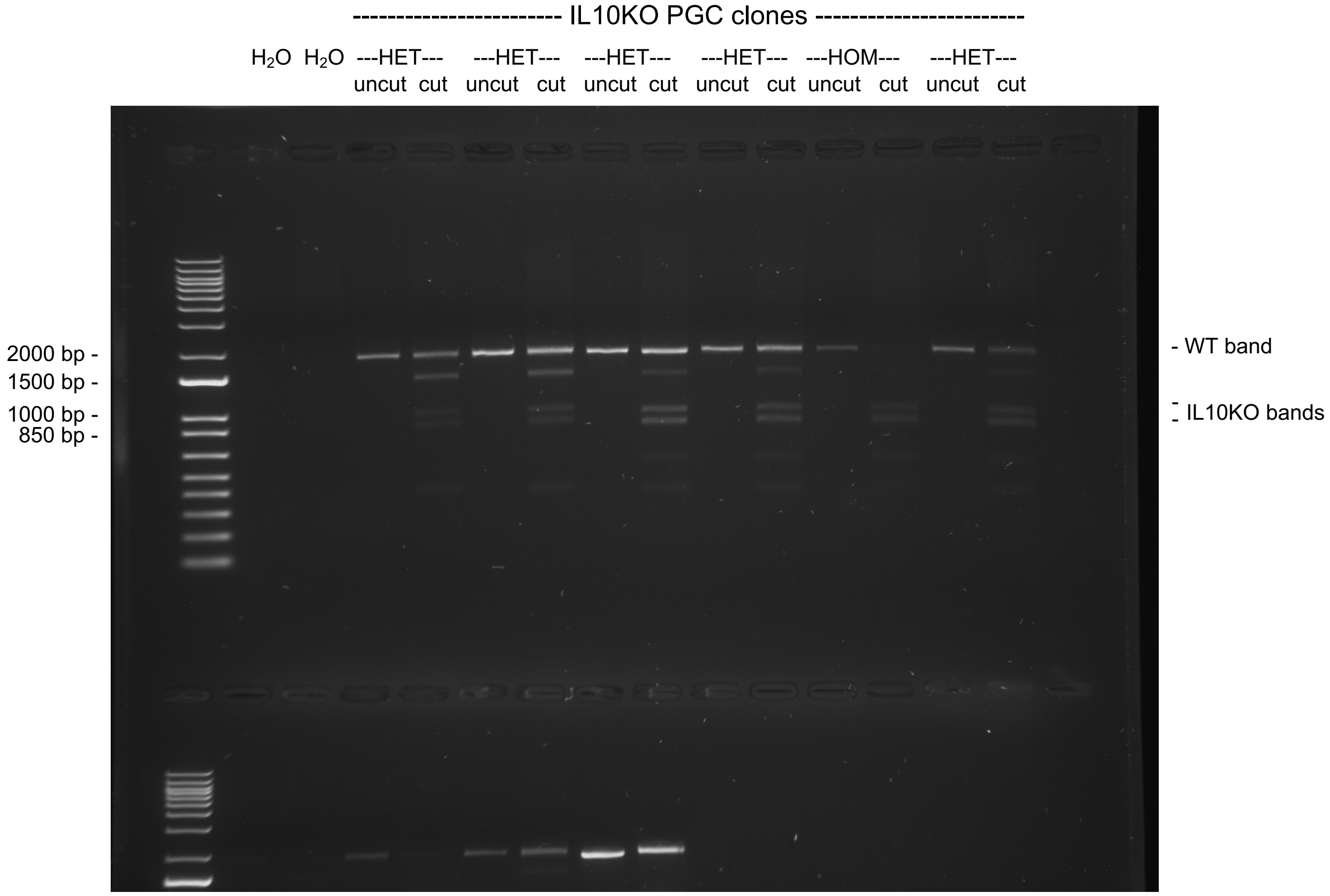

Figure 1—figure supplement 4—source data 1—panel A: Annotated raw, unedited and uncropped agarose gel image corresponding to Figure 1—figure supplement 4A, with the relevant bands clearly labelled.

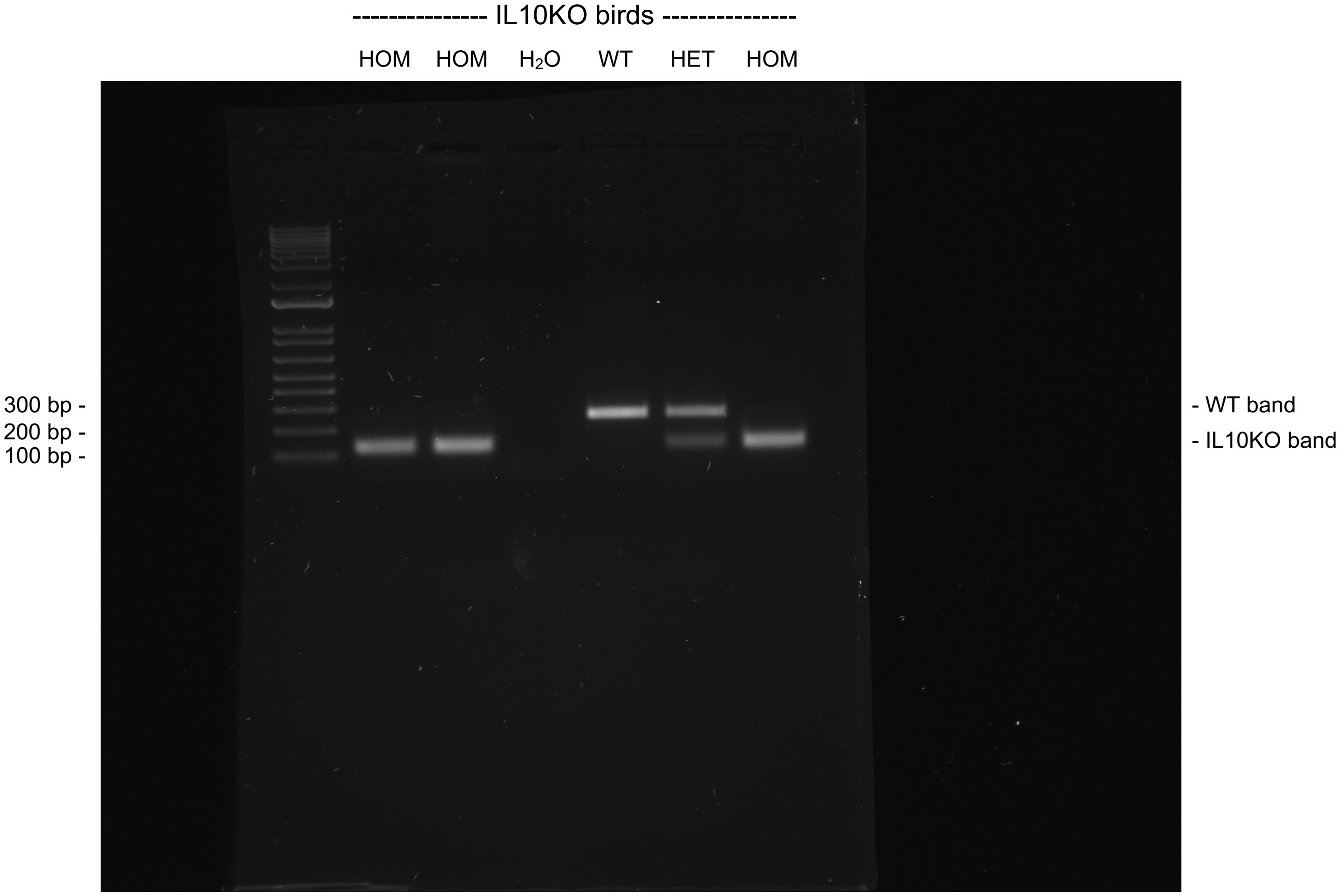

Figure 1—figure supplement 4—source data 1—panel B: Annotated raw, unedited and uncropped agarose gel image corresponding to Figure 1—figure supplement 4B, with the relevant bands clearly labelled.

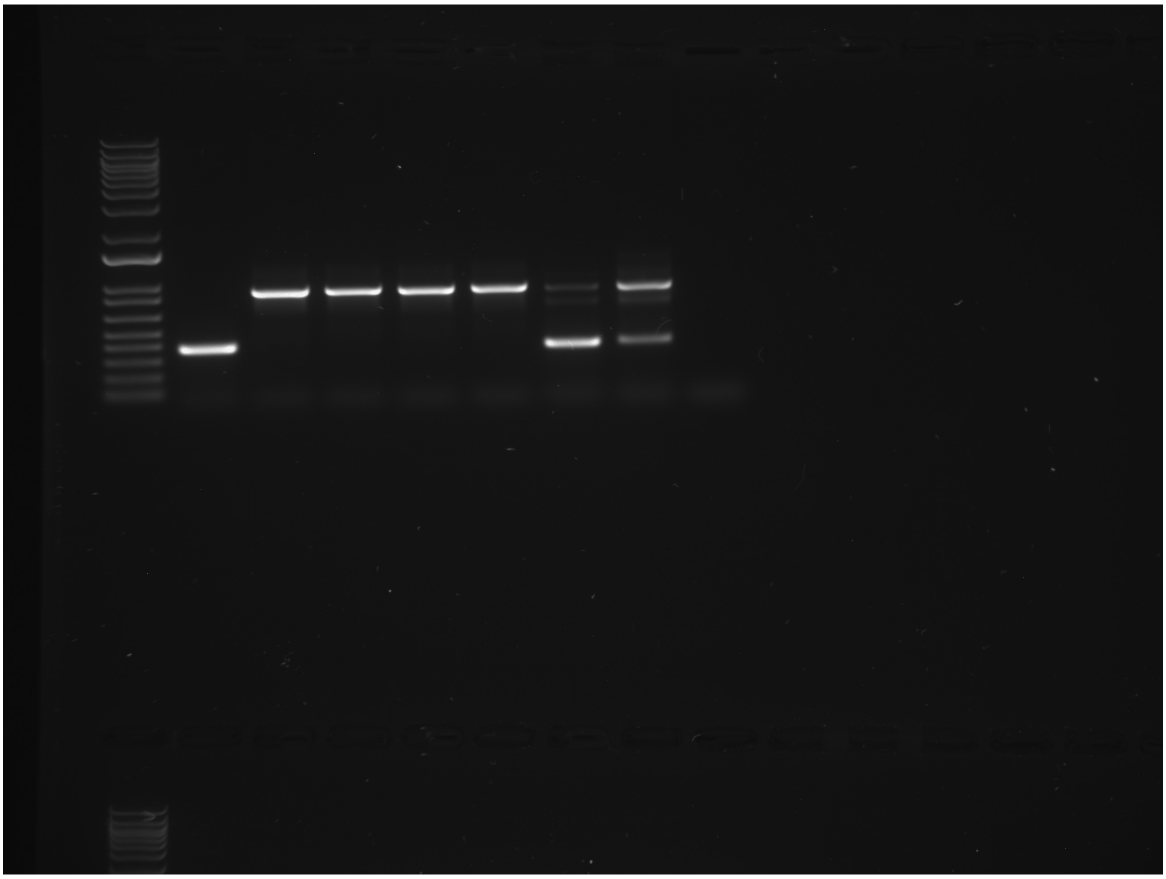

Figure 1—figure supplement 4—source data 1—panel C: Annotated raw, unedited and uncropped agarose gel image corresponding to Figure 1—figure supplement 4C, with the relevant bands clearly labelled.

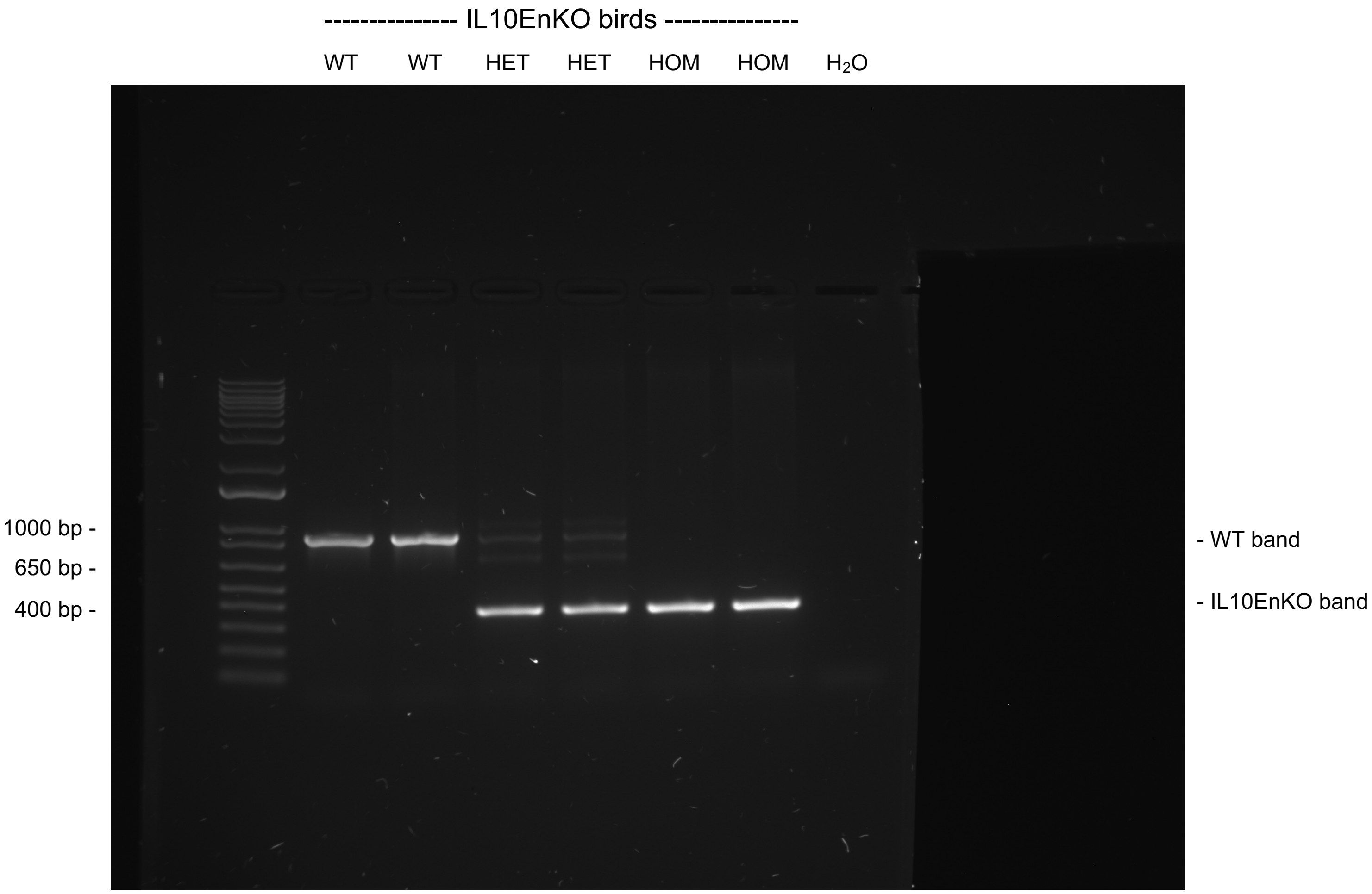

Figure 1—figure supplement 4—source data 1—panel D: Annotated raw, unedited and uncropped agarose gel image corresponding to Figure 1—figure supplement 4D, with the relevant bands clearly labelled.
