# Supplementary figures and images for: Interleukin 10 controls the balance between tolerance, pathogen elimination, and immunopathology in birds
Source: eLife. 2025 Oct 16;14:RP106252. doi: 10.7554/eLife.106252 (PMC12530801; doi:10.7554/eLife.106252)

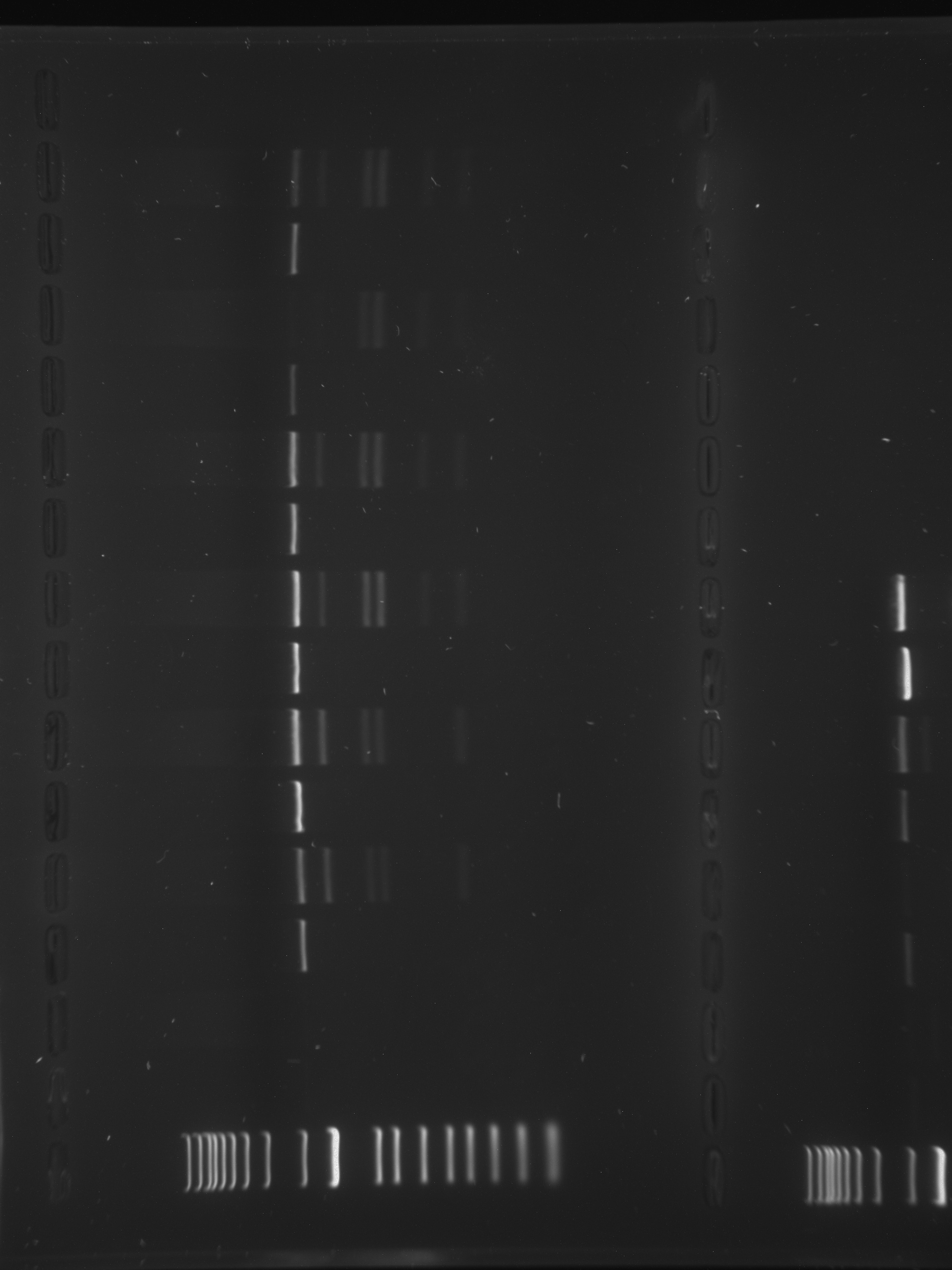

Supplement: Figure 1—figure supplement 4—source data 2. [file elife-106252-fig1-figsupp4-data2.zip › Figure 1-figure supplement 4-source data 2/Figure 1-fig suppl 4A (DM300120_AVRII-00001).jpg]

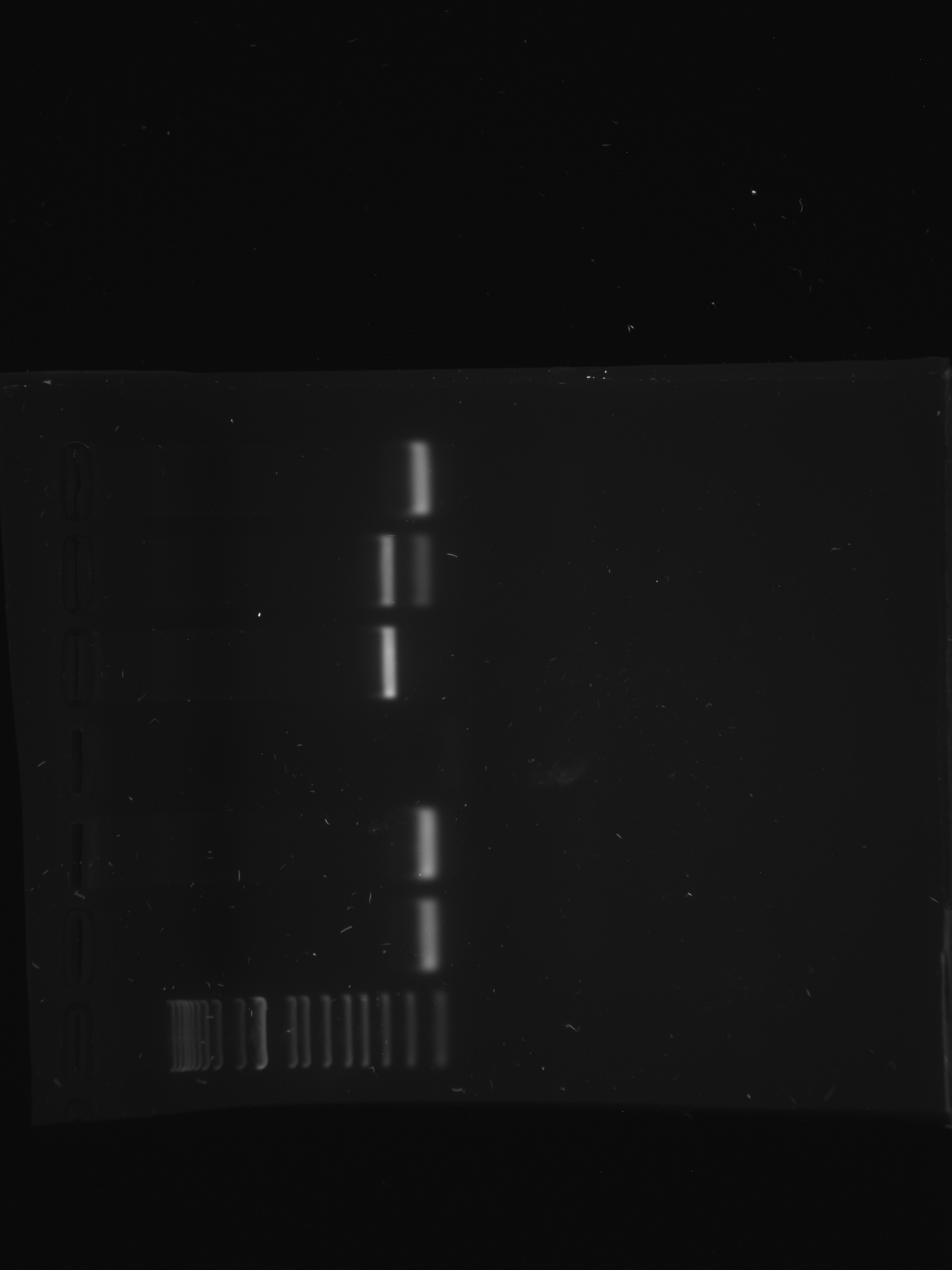

Supplement: Figure 1—figure supplement 4—source data 2. [file elife-106252-fig1-figsupp4-data2.zip › Figure 1-figure supplement 4-source data 2/Figure 1-fig suppl 4B (DM21042022_AVRII-00001).jpg]

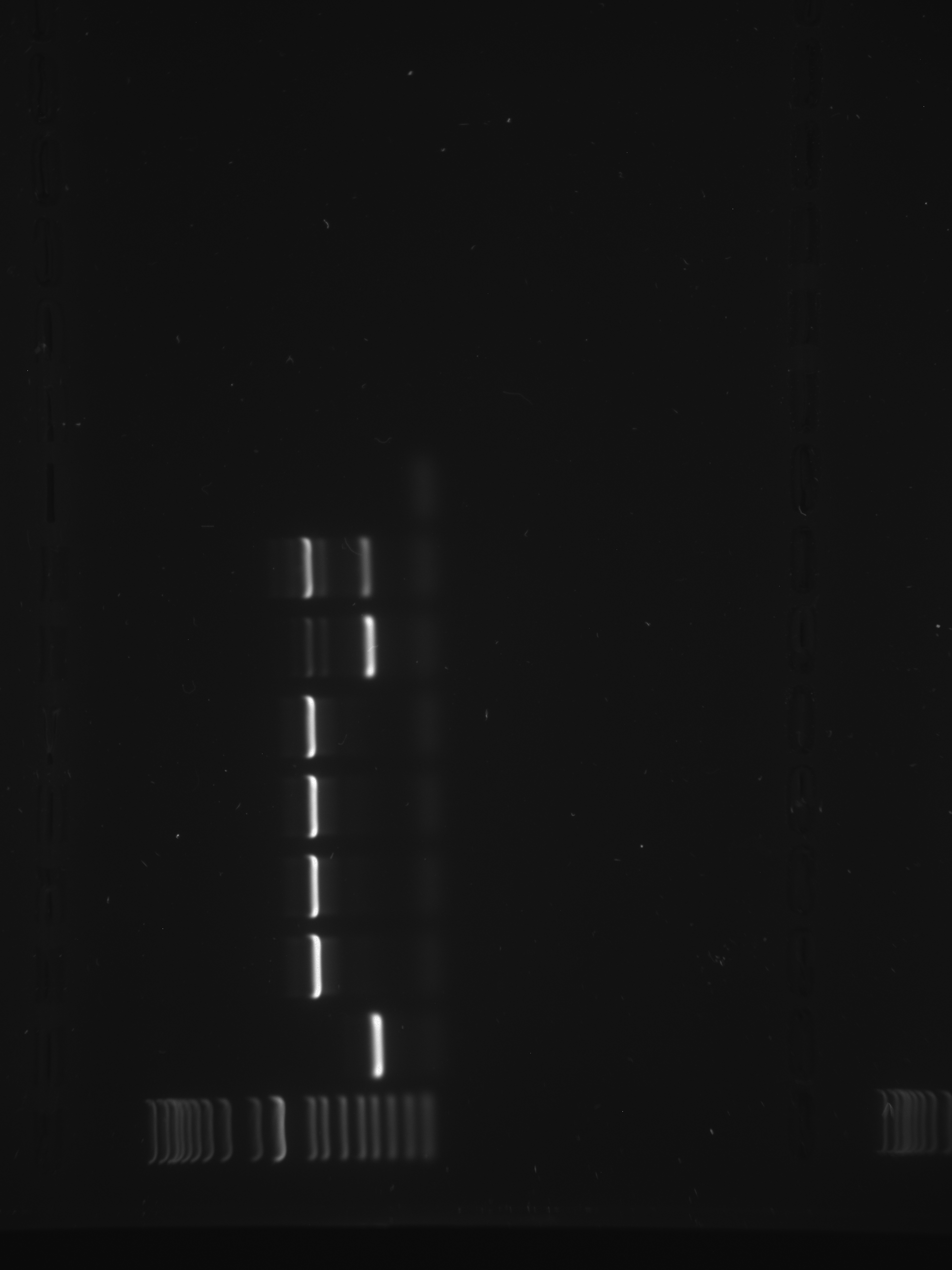

Supplement: Figure 1—figure supplement 4—source data 2. [file elife-106252-fig1-figsupp4-data2.zip › Figure 1-figure supplement 4-source data 2/Figure 1-fig suppl 4C (DM261119_FEM5231-00003).tif]

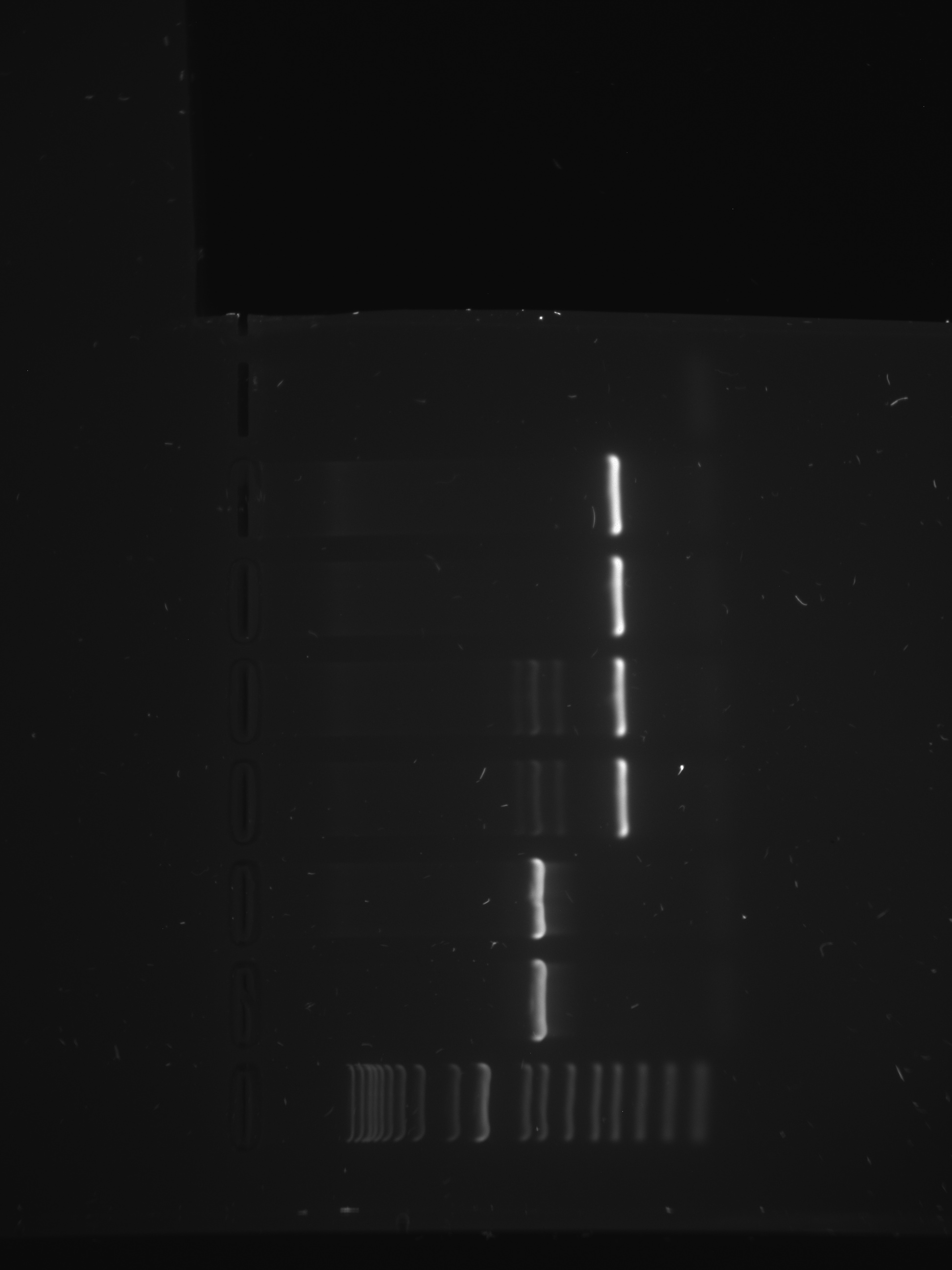

Supplement: Figure 1—figure supplement 4—source data 2. [file elife-106252-fig1-figsupp4-data2.zip › Figure 1-figure supplement 4-source data 2/Figure 1-fig suppl 4D (DM260422_ENHANCER-00001).jpg]
