# Supplementary material for: Interleukin 10 controls the balance between tolerance, pathogen elimination, and immunopathology in birds
Source: eLife. 2025 Oct 16;14:RP106252. doi: 10.7554/eLife.106252 (PMC12530801; doi:10.7554/eLife.106252)
Supplement: Supplementary file 1. — n.d.: not determined. [file elife-106252-supp1.docx]

**Supplementary File 1**: Number of IL10KO and IL10EnKO WT, HET and HOM chicks hatched in the NARF SPF chicken facility in the first (G1) and second (G2) generations

| **Line** | **Parental cross** | **Generation** | **Total chicks hatched** | **Number of chicks per genotype** | | | |
| --- | --- | --- | --- | --- | --- | --- | --- |
|  |  |  |  | **WT** | **HET** | **HOM** | **n.d.** |
| IL10KO | iCaspase9 surrogate host (G0) x RIR | G1 | 102 | 0  (0%) | 102  (100%) | 0  (0%) | 0  (0%) |
|  | HET (G1) x HET (G1) | G2 | 435 | 105  (24%) | 209  (48%) | 94  (22%) | 27  (6%) |
| IL10EnKO | iCaspase9 surrogate host (G0) x RIR | G1 | 102 | 0  (0%) | 102  (100%) | 0  (0%) | 0  (0%) |
|  | HET (G1) x HET (G1) | G2 | 246 | 62  (25%) | 104  (42%) | 67  (27%) | 13  (5%) |
