# Supplementary material for: Interleukin 10 controls the balance between tolerance, pathogen elimination, and immunopathology in birds
Source: eLife. 2025 Oct 16;14:RP106252. doi: 10.7554/eLife.106252 (PMC12530801; doi:10.7554/eLife.106252)
Supplement: Supplementary file 2. [file elife-106252-supp2.docx]

**Supplementary File 2**: Routine vaccination schedule in the NARF conventional chicken facility

| **Age at vaccination** | **Vaccines** | **Type of vaccine** | **Route of administration** | **Pathogens** |
| --- | --- | --- | --- | --- |
| Day old | Prevexxion RN | Live | Intra-muscular injection | Marek’s disease virus (MDV) |
| 5-7 days | Evant | Live | Drinking water | Avian coccidiosis virus (ACV) |
| 3-5 weeks | Nobilis Gumboro D78 | Live | Drinking water | Infectious bursal disease virus (IBDV) |
| 3-5 weeks | Poulvac IB Primer | Live | Drinking water | Infectious bronchitis virus (IBV) |
| 3-5 weeks | Nobilis ND Clone 30 | Live | Drinking water | Newcastle disease virus (NDV) |
| 6-9 weeks | Poulvac ILT | Live | Eye drop | Infectious laryngotracheitis virus (ILTV) |
| 6-12 weeks | Poulvac AE | Live | Drinking water | Avian encephalomyelitis virus (AEV) |
| 8-12 weeks | AviPro Thymovac | Live | Drinking water | Chicken anaemia virus (CAV) |
| 9-14 weeks | Nobilis IB Ma5 | Live | Drinking water | Infectious bronchitis virus (IBV) |
|  | Nobilis IB 4-91 | Live | Drinking water |  |
| 9-14 weeks | Nobilis ND Clone 30 | Live | Drinking water | Newcastle disease virus (NDV) |
| 16 weeks and over | Nobilis RT+IBmulti+ND  +EDS | Inactivated | Intra-muscular injection | Avian rhinotracheitis virus (ARV) |
|  |  |  |  | Infectious bronchitis virus (IBV) |
|  |  |  |  | Newcastle disease virus (NDV) |
|  |  |  |  | Duck adenovirus, the agent of egg drop syndrome (EDS) |
