# Supplementary material for: Interleukin 10 controls the balance between tolerance, pathogen elimination, and immunopathology in birds
Source: eLife. 2025 Oct 16;14:RP106252. doi: 10.7554/eLife.106252 (PMC12530801; doi:10.7554/eLife.106252)
Supplement: Supplementary file 3. [file elife-106252-supp3.docx]

**Supplementary File 3**: PCR primer sequences and expected fragment sizes

| **Primer name** | **Sequence 5’-3’** | **Expected fragment sizes** | |
| --- | --- | --- | --- |
|  |  | **WT allele** | **Edited or transgenic allele** |
| Myco3_14709 | GCG GTG TGT ACA AGA CCC GA | ~500 bp  (MS and MG) | N/A |
| Myco5_14712 | TGC CTG AGT AGT ACA TTC GC |  |  |
| Myco5_14713A | CGC CTG AGT AGT ATG CTC GC |  |  |
| IL10_Exon1_F1 | TAACCCCACGAAACAGAAGGAG | 243 bp | ~115 and 128 bp (after AvrII digestion) |
| IL10_Exon1_R1 | ATCAAGGGCAGCAGCAGAATAA |  |  |
| IL10_Exon1_F4 | AAGATGTGCTTTATGCAGGCAG | 1826 bp | ~855 and 965 bp (after AvrII digestion) |
| IL10_Exon1_R4 | GGGAAGCATGCAGAACTGAC |  |  |
| IL10-Enhancer_F2 | GGGTCGTCCCACACTTTCCT | 912 bp | ~380 bp |
| IL10-Enhancer_R2 | GAGTCAAGCTGCAAATTCCGAC |  |  |
| LTR_U3_F | TCCTCTGGTTTCCCTTTCGC | 320 bp | 400 bp |
| 235_F | CAGCCCACCCATCTCATCTC |  |  |
| 235_R | GACATTTCCTGCCTCCCCAG |  |  |
