# Supplementary material for: Interleukin 10 controls the balance between tolerance, pathogen elimination, and immunopathology in birds
Source: eLife. 2025 Oct 16;14:RP106252. doi: 10.7554/eLife.106252 (PMC12530801; doi:10.7554/eLife.106252)
Supplement: Supplementary file 4. — Note the three substituted nucleotides (red, lowercase) and AvrII restriction site (CCTAGG, underlined) in the ssODN sequence. [file elife-106252-supp4.docx]

**Supplementary File 4**: Guide RNA and ssODN sequences. Note the 3 substituted nucleotides (red, lowercase) and AvrII restriction site (CCTAGG, underlined) in the ssODN sequence.

| **Target** | **Name** | **Sequence 5’-3’** |
| --- | --- | --- |
| *IL10* exon 1 | ggIL10_exon1_g3 | GCAGCAGCTCAGAGAAGTGC |
|  | IL10_exon1_HDRoligo2 | ACCTGCTGCCAAGCCCTGTTGCTGCTGCTGGCTGCATGCACCCTGCCTGCCCACTGCTTGGAGCCCACCT**agg**TGCACTTCTCTGAGCTGCTGCCCGCCCGGCTGCGGGAGCTGAGGGTGAAGTTTGAGGAAATTAAGGACTA |
| *IL10* putative enhancer region | IL10_enhancer_guide1 | GTTTCGTAGCGGGTGAATGA |
|  | IL10_enhancer_guide2 | GTGCAGGGCAGTTTCCTTTG |
