# Supplementary material for: Interleukin 10 controls the balance between tolerance, pathogen elimination, and immunopathology in birds
Source: eLife. 2025 Oct 16;14:RP106252. doi: 10.7554/eLife.106252 (PMC12530801; doi:10.7554/eLife.106252)
Supplement: Supplementary file 5. [file elife-106252-supp5.docx]

**Supplementary File 5**: NARF SPF screening

| **Pathogens** | **ELISA** | **PCR** |
| --- | --- | --- |
| 1. Avian adenovirus (FAV-1) | X |  |
| 2. Avian encephalomyelitis virus (AEV) | X |  |
| 3. Avian influenza virus (AIV) | X |  |
| 4. Avian leukosis virus (ALV) | X |  |
| 5. Avian pneumovirus (APV) | X |  |
| 6. Avian reovirus (REO) | X |  |
| 7. Avian reticuloendotheliosis virus (REV) | X |  |
| 8. Chicken anaemia virus (CAV) | X |  |
| 9. Duck adenovirus, the agent of egg drop syndrome (EDS) | X |  |
| 10. Infectious bronchitis virus (IBV) | X |  |
| 11. Infectious bursal disease virus (IBDV) | X |  |
| 12. Infectious laryngotracheitis virus (ILTV) | X |  |
| 13. Marek’s disease virus (MDV) |  | X |
| 14. Mycoplasma gallisepticum (MG) | X |  |
| 15. Mycoplasma synoviae (MS) | X |  |
| 16. Newcastle disease virus (NDV) | X |  |
| 17. Salmonella gallinarum & pullorum | X |  |
